# Urbanization processes drive divergence at the major histocompatibility complex in a common waterbird

**DOI:** 10.7717/peerj.12264

**Published:** 2021-10-05

**Authors:** Ewa Pikus, Radosław Włodarczyk, Jan Jedlikowski, Piotr Minias

**Affiliations:** 1 Department of Biodiversity Studies and Bioeducation, University of Łódź, Łódź, Poland; 2 Biological and Chemical Research Centre, University of Warsaw, Warsaw, Poland

**Keywords:** Local adaptation, Eurasian coot, Major histocompatibility complex, Population differentiation, Urbanization, MHC, Urban evolution

## Abstract

Urban sprawl is one of the most common landscape alterations occurring worldwide, and there is a growing list of species that are recognised to have adapted to urban life. To be successful, processes of urban colonization by wildlife require a broad spectrum of phenotypic (*e.g*., behavioural or physiological) adjustments, but evidence for genetic adaptations is much scarcer. One hypothesis proposes that different pathogen-driven selective pressures between urban and non-urban landscapes leads to adaptations in host immune genes. Here, we examined urbanization-related differentiation at the key pathogen-recognition genes of vertebrate adaptive immunity-the major histocompatibility complex (MHC)-in a common waterbird, the Eurasian coot (*Fulica atra*). Samples were collected from an old urban population (established before the 1950s), a new urban population (established in the 2000s), and two rural populations from central Poland. We found strong significant divergence (as measured with Jost’s D) at the MHC class II between the old urban population and the remaining (new urban and rural) populations. Also, there was a moderate, but significant divergence at the MHC between the new urban population and two rural populations, while no divergence was found between the two rural populations. The total number of MHC alleles and the number of private (population-specific) MHC alleles was lower in old urban populations, as compared to the rural ones. These patterns of differentiation at the MHC were not consistent with patterns found for neutral genetic markers (microsatellites), which showed few differences between the populations. Our results indicate that MHC allele composition depended on the level of anthropogenic disturbance and the time which passed since urban colonization, possibly due to the processes of genotype sorting and local adaptation. As such, our study contributes to the understanding of genetic mechanisms associated with urbanization processes in wildlife.

## Introduction

For the first time in our history more than a half of the world’s human population lives in cities, and by 2030 every third resident is expected to live in a city of at least a half-million people (Camacho-Valdez et al., 2009; McDonald, Kareiva & Forman, 2008). Such intense urbanization entails huge investments in urban infrastructure, which significantly changes the natural landscape and, at the same time, creates novel urban ecosystems and novel ecological niches (Aouissi et al., 2017; Croci, Butet & Clergeau, 2008; Green et al., 2016). Many animal species may not be able to adapt and reproduce in highly altered landscapes outside of their natural niches, particularly those less plastic and more ecologically specialized, and thus they are often excluded from urban environments (Concepción et al., 2015; Luniak, 2004). Despite the challenges posed by urban environments, more and more animal species are adapting to new anthropogenic environmental conditions and colonizing cities-overcoming the ecological, demographic, and behavioural barriers (Luniak, 2004). Urban areas may be an attractive habitat alternative for species that can take advantage of these novel ecosystems, including benefiting from access to anthropogenic food, milder climate during the winter, and lower pressure from natural predators (Davis, Malas & Minor, 2014; Luniak, 2004; Minias & Janiszewski, 2016). Conversely, the use of urban resources by wild animals often comes at some costs (*e.g*., exposure to novel pathogens).

Anthropogenic environmental change may promote the emergence of novel pathogens through the transportation and introduction of infectious agents or hosts to new environments, through manipulation of local ecosystems which can favour the proliferation or prolonged survival of infectious agents or by facilitating new host–pathogen interactions (Miller et al., 2002). Relatively poor biodiversity of urban areas leads to the simplified regulation processes of animal communities (Adams, Van Druff & Luniak, 2005), and thus, habitat-related differences in pathogen pressure may pose a challenge during the process of urban colonization. Urbanization generally reduces the abundance and diversity of parasites, but on the other hand parasite or pathogen transmission can, in some cases, increase among urban-adapted hosts (Bradley & Altizer, 2007; Brearley et al., 2013). For example, a recently established urban population of dark-eyed juncos (*Junco hyemalis*) was found to host significantly higher numbers of ectoparasites and had more pox lesion scars compared to non-urban populations from montane habitats (Whittaker et al., 2012). Additionally, urban wildlife can be exposed to novel parasites found in the faeces of domestic animals, such as the infectious oocytes of the protozoan parasite *Toxoplasma gondii*, the causative agent of toxoplasmosis (Bradley & Altizer, 2007; Frenkel et al., 1995). The differences in the composition of pathogen faunas between urban and non-urban areas may require necessary adaptations of the immune system (Alcaide et al., 2008; Watson et al., 2017). Urban bullfinches (*Loxigilla barbadensis*) are known to have enhanced immunocompetence compared to non-urban conspecifics (Audet, Ducatez & Lefebvre, 2016) and transcriptomic analysis of great tits (*Parus major*) has provided strong evidence for the upregulation of adaptive immune responses in urban individuals (Watson et al., 2017). However, genetic variation in urban colonizing wildlife may not only be driven by selective processes, such as local adaptation (Loiseau et al., 2009), but also by stochastic processes, like genetic drift (Lourenço et al., 2017). Genetic bottlenecks may occur during the establishment of novel urban populations (*i.e*., the founder effect), which can result in a low level of genetic diversity, including the diversity of the immune receptors responsible for antigen recognition (Alcaide, 2010; Sutton et al., 2011; Strand et al., 2012).

Two families of pathogen-recognition genes play a key role in innate and adaptive immunity: toll-like receptors (TLR) and the major histocompatibility complex (MHC) (Schenten & Medzhitov, 2011). In general, TLRs are responsible for identifying conservative antigens (pathogen-associated molecular patterns, PAMP) which are characteristic of a wide spectrum of pathogens and parasites (Barton & Medzhitov, 2020). In turn, MHC molecules activate adaptive immune response (Ekblom et al., 2007) by presenting specific intercellular (MHC class I) or extracellular (MHC class II) antigens to T cells (Piertney & Oliver, 2006). MHC genes are the most polymorphic genes in vertebrates and the majority of polymorphism is concentrated in the peptide-binding region (PBR) which binds peptides originating from pathogen processing (*i.e*., from the proteasomal proteolysis or lysosomal hydrolysis of pathogens) (Hess & Edwards, 2002; Hughes & Yeager, 1998; Münz, 2012). Even minor amino acid alterations in PBR may lead to large differences in the repertoire of the peptides recognized and, as a consequence, change the spectrum of the pathogens against which an organism effectively activates the immune response (Dionne et al., 2007). In general, pathogen-driven balancing selection-acting through the mechanisms of heterozygote advantage, negative frequency-dependent selection, and fluctuating selection-maintains extraordinary diversity of MHC in natural populations; unlike the relatively low variable and structurally conservative TLRs (Barton & Medzhitov, 2020; Borghans, Beltman & De Boer, 2004). Thus, MHC allelic composition within populations should quickly respond *via* local adaptation to changes in the composition of pathogens and parasite fauna (Ekblom et al., 2007; Miller, Allendorf & Daugherty, 2010).

So far, the strongest evidence for associations between immune genes and urbanization comes from mammals. For example, significant genetic variation at neutral and immune gene (MHC and TLR) linked microsatellite loci was found between populations of bobcats (*Lynx rufus*) whose habitats differed in urbanization level (Serieys et al., 2015). Red foxes (*Vulpes vulpes*) that colonized Zurich, Switzerland, showed depleted neutral and functional (immune-linked) genetic diversity following a founder event (DeCandia et al., 2019a). Also, significant differentiation between fox populations separated by natural and anthropogenic barriers was found at both types of markers, with evidence of selection acting on MHC-linked markers (DeCandia et al., 2019a). In contrast to the older colonization of Zurich by red foxes, there was little support for balancing selection maintaining diversity at the MHC-linked loci of coyotes (*Canis latrans*) after the recent colonization of the New York metropolitan area, USA (DeCandia et al., 2019b). Differentiation of MHC genes has been detected in birds at the landscape level, but little research has tested for the effects of urbanization. For example, significant between-population differentiation at the MHC class II genes contrasted with the relatively homogeneous distribution of microsatellite alleles in the lesser kestrel (*Falco naumanni*; Alcaide et al., 2008) and great snipe (*Gallinago media*; Ekblom et al., 2007). These contrasting patterns were primarily attributed to variation in habitat structure and processes of local adaptation to site-specific pathogen faunas (Alcaide et al., 2008; Ekblom et al., 2007).

Our study aims to examine the differentiation and diversity of MHC genes in a common waterbird, the Eurasian coot (*Fulica atra*, Rallidae, Gruifomes), from areas that varied in urbanization level. For this purpose, we genotyped MHC class II genes in four populations of the Eurasian coot from central Poland: (1) an old urban population (Warszawa; urban area colonized by coots before 1950); (2) a new urban population (Łódź; urban area colonized by coots at the beginning of the 2000s); and (3) two non-urban populations (Sarnów and Żeromin). Since the process of urban colonization by the Eurasian coot is still in progress in central Europe, the species may be considered a good model to study genetic adaptations across urban and non-urban populations with different histories. Here, we test whether urbanization processes are related to changes in the MHC gene pool and if they lead to reduced immunogenetic diversity at the population level. We hypothesized that differences in urbanization level altered the allelic composition of MHC genes between urban and non-urban Eurasian coot populations, with more pronounced differences observed in the old *versus* new urban population. We additionally hypothesized that the process of urban colonization would be associated with reduced MHC diversity, either due to local adaptation (no parallel reduction in neutral diversity expected) or demographic history and genetic drift (parallel reduction in neutral diversity expected).

## Materials and Methods

### Study area

**Figure 1:**
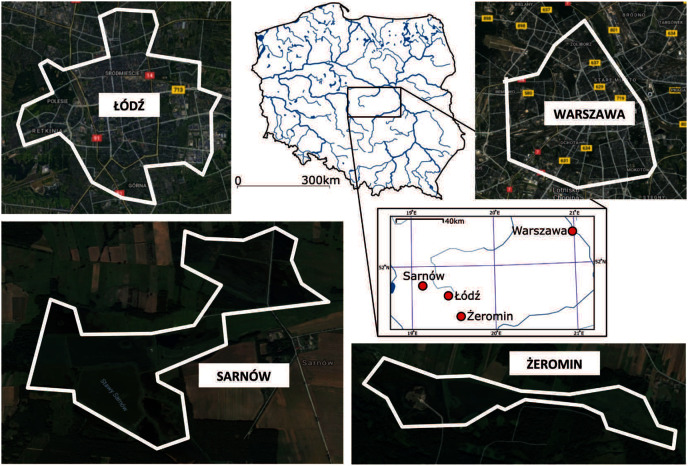
Location and land cover of sampling sites. Borders of sampling areas are marked with white lines. Satellite map data © 2021 Google, CNES/Airbus, MGGP Aero, Maxar technologies.

Data were collected in two urban (Warszawa and Łódź) and two rural (Sarnów and Żeromin) populations of Eurasian coot from central Poland during 2012–2018 (Fig. 1). Warszawa (52.259° N, 21.020° E) is the largest city in Poland in terms of population size (1.78 million inhabitants in 2020; according to data by Statisctics Poland, Poland, https://stat.gov.pl/en/), and area (517.24 km^2^); it is also characterized by high population density (3,462 people/km^2^ in 2020). The city was colonized by coots in the mid-20th century, with a stable breeding population of coots being reported in highly urbanized parts of Warszawa as early as in the 1960s (Luniak, Kalbarczyk & Pawłowski, 1964). During the 1980s there were already 190–210 breeding pairs (Luniak et al., 2001) in the city. The relatively early establishment of the urban population in Warszawa was likely facilitated by the presence of a large river (Vistula) in the city centre. Łódź (51.757° N, 19.493° E) is the third largest city in Poland in terms of population size (672,185 inhabitants in 2020; Statisctics Poland, Poland), and its administrative area covers 293.25 km^2^ with a population density of 2,309 people/km^2^ in 2020. The urban areas of Łódź were colonized by coots relatively recently, with only ~20 coot nesting sites being recorded exclusively on the outskirts of Łódź during surveys in 1994–2002 (Janiszewski, Wojciechowski & Markowski, 2009). Since then, several locations in the city centre have been colonized. During the period of this study, the coot population size in Łódź was estimated at ~70 breeding pairs, including ~40 pairs breeding in the city centre. Because of evident differences in the timing of colonization, Warszawa and Łódź populations are henceforth referred to as old and new urban populations, respectively. In the natural environment, coots inhabit a variety of standing waterbodies, such as lakes, field ponds, oxbow lakes, but also in the reservoirs of artificial origin, including fish ponds, clay pit ponds, peat lakes, storage reservoirs, or channels. Rural populations of coots were located at fish ponds in Sarnów (51.851° N, 19.109° E) and Żeromin (51.617° N, 19.607° E), which provided semi-natural nesting habitat with extensive reed areas (Fig. 1). Trespassing of unauthorized personnel was restricted at both sites, resulting in low human presence and a relatively low degree of anthropogenic pressure.

### Sample collection and DNA isolation

Blood samples were taken during the reproductive season from 20–30 adult breeding individuals per population (*n* = 103 individuals in total). Birds were caught with noose traps put in the nests and made from monofilament nylon. Several birds were caught by hand (exclusively in urban populations). Approximately 75 μl of blood was taken from a tarsal vein from each bird and stored in 96% ethanol at 2 °C until further analysis. Each bird was ringed and marked with a plastic neck collar, which allowed us to avoid recapturing the same individuals. Genomic DNA was isolated from blood samples with GeneJET Genomic DNA Purification Kit (Fermentas, Thermo Fisher Scientific, Waltham, MA, USA) following the manufacturer’s protocol. All methods were carried out in accordance with the current laws of Poland (Act on Nature Conservation from 16 April 2004, Journal of Laws from 2004, No. 92, item 880) and were approved by the Local Bioethical Commission for Experiments on Animals in Łodź (nos 40/ŁB 620/2012 and 15/ŁB/2016).

### Microsatellite genotyping and variation

To assess the level of neutral genetic variation in our study populations, we used ten microsatellite loci (Table S1). Nine microsatellite markers were originally developed for other Rallidae species: Tm18, Tm20, Tm27, Tm38 (Tasmanian native-hen, *Tribonyx mortierii*) (Buchan, 2000), Kira8, Kira9, Kira10, Kira16 (king rail, *Rallus elegans*) (Brackett et al., 2013), and B106 (black rail, *Laterallus jamaicensis*) (Spautz, Nur & Stralberg, 2005). The last marker, TG03002, was highly conserved and amplified successfully in a wide range of passerine and non-passerine bird species (Dawson et al., 2010). PCR amplifications followed original protocols and were conducted in a total volume of 20 µl containing ten µl of 2X DreamTaq PCR Master Mix (DreamTaq DNA Polymerase, 2X DreamTaq buffer with four mM MgCl_2_, and 0.4 mM of each dNTP) (ThermoFisher Scientific, Waltham, MA, USA), eight µL of deionized water, 0.5 µL of each primer (final concentration of 1.25 µM) and one µL of genomic DNA solution (20–80 ng of DNA). Fragment size analysis was conducted with ABI/Hitachi 3,500 (Applied Biosystems, Foster City, CA, USA) sequencer. The size of alleles was assessed with GeneScan TM 600 LIZ Standard (Applied Biosystems, Foster City, CA, USA) using Geneious 7.1.7 software (Biomatters, Auckland, New Zealand). All loci were successfully genotyped in all individuals. To exclude any major genotyping errors allele calling was checked twice and genotyping of technical replicates showed high (>95%) reproducibility of alleles.

We found no deviation from the Hardy–Weinberg equilibrium at any locus in any population (all *p* > 0.05 after Bonferroni correction), as assessed with the exact tests (Guo & Thompson, 1992) that were run with the Markov chain method (chain length: 1,000,000; dememorization: 1,00,000) in Arlequin 1.3.5.2 software (Excoffier & Lischer, 2010). Also, no evidence for linkage disequilibrium was found between any pair of loci (all *p* > 0.05 after Bonferroni correction), as tested with FSTAT 2.9.3 software (Goudet, 1995). The frequency of null alleles was relatively low (max. 0.052 at Kira-16), as checked with Cervus 3.0.3 (Kalinowski, Taper & Marshall, 2007). Micro-Checker 2.2.3 (Van Oosterhout et al., 2004) did not indicate genotyping errors due to null alleles, short allele dominance (large allele dropout), or stuttering. The observed and expected heterozygosity was calculated with GeneAlEx v.6.5 software (Peakall & Smouse, 2006; Smouse & Peakall, 2012).

### MHC genotyping

MHC genotyping focused on class II genes, which were previously reported to show high level of polymorphism in natural coot populations (Alcaide et al., 2014). For amplifications we used species-specific primers, Fuat-Ex2Fw (5′-CTGACCRGCCTCCCTGCA-3′) and Fuat-Ex2Rv (5′-TTGTGCCAYACACCCACC-3′), which amplify the entire MHC class II exon 2 (270 bp), binding with the neighbouring regions of intron 1 and 2 (Alcaide et al., 2014). Our targeted region (exon 2) codes for one of two domains that form the peptide-binding groove of the MHC molecule and, thus, have a direct impact on antigen recognition. PCR amplifications were done in a total volume of 20 µl containing ten µl of 2X HotStarTaq Plus MasterMix Kit (HotStarTaq Plus DNA Polymerase, PCR Buffer with 0 mM MgCl_2_, and 400 μM of each dNTP) (Qiagen, Venlo, Netherlands), eight µl of deionized water, 0.5 µl of each primer (final concentration of 1.25 µM), and one µl of genomic DNA solution (20–80 ng of DNA). To allow identification of samples, amplifications were completed using fusion primers containing Illumina Nextera Transposase adapter sequences (Illumina Corp., San Diego, CA, USA), seven-bp barcodes that indicated sample identity, and original MHC primers. Following Alcaide et al. (2014), conditions for PCR reactions consisted of an initial denaturation (95 °C for 5 min) followed by 24 cycles of denaturation (95 °C for 60 s), annealing (60 °C for 40 s) and elongation (72 °C for 60 s), and a final extension (72 °C for 10 min). The number of PCR cycles was set to 24 to reduce the risk of producing chimeras. All PCR products were purified, and their concentration was assessed with a Quant-iT PicoGreen dsDNA marking kit (Thermo Fisher Scientific, Waltham, MA, USA). A library was prepared from equimolar quantities of PCR products using NEB-Next DNA Library Prep Master Mix Set for Illumina (New England Biolabs, Ipswich, MA, USA) and sequenced on the 2 × 250 bp Illumina MiSeq platform.

### Processing of Illumina data and MHC allele validation

Raw Illumina MiSeq data were processed using Amplicon Sequencing Analysis Tools (AmpliSAT) web server (Sebastian et al., 2016) and processing algorithms recommended by Biedrzycka et al. (2017). To merge pair-ended reads we used FLASH algorithm (Magoc & Salzberg, 2011) with optimum overlapping parameters (determined based on amplicon data), as implemented in AmpliMERGE tool. Then, AmpliSAS tool was used for de-multiplexing, clustering, and filtering of reads. Default parameters for Illumina data were used for clustering (1% substitution errors, 0.001% indel errors, and 25% minimum dominant frequency), while in the filtering step we discarded chimeras and sequences that had less than 3% frequency. Minimum amplicon depth was set to 300 reads, while maximum amplicon depth was set to 5,000 reads (due to processing limitations of AmpliSAS). Before data processing, the average sequencing (amplicon) depth was 1,706 ± 73 [SE] per sample, while the average number of reads for validated alleles was 1,398 ± 64 [SE] reads per sample. Reproducibility of alleles was assessed using 25 technical replicates (independent PCR amplifications from the same individuals). All reads had 273 or 276 bp length and the difference in sequence length was due to a single-codon deletion, previously reported for MHC class II exon 2 of the Eurasian coot (Alcaide et al., 2014). All validated alleles were aligned in Geneious v10.0.5 (Biomatters Ltd., Auckland, New Zealand). Intron regions (two codons) were removed from the alignments, retaining full length (267/270 bp) MHC class II exons 2 sequences, which are referred to as alleles in all further analyses.

### Statistical analyses

Basic measures of allelic diversity within populations (*i.e*., total number of alleles and the number of private alleles per population) were calculated for MHC and microsatellite loci using GeneAlEx v.6.5 software. For microsatellite loci we also calculated mean observed heterozygosity for each population using the same software, while for the MHC we calculated population-specific sequence polymorphism measures (number of segregating sites, number of nucleotide differences, nucleotide diversity) using DnaSP v6.10.03 (Rozas et al., 2017). We used rarefaction to standardize all population-specific measures of allelic diversity for a population size of 20 individuals, which was the minimum sample size available for any of the study populations (Tables S2, S3 in File S2). Differences in the individual MHC diversity (*i.e*., number of alleles per individual) and individual microsatellite heterozygosity between populations were tested with general linear mixed models (GLMM) in *glmmADMB* package (Skaug et al., 2012) developed for the R v3.6.3 statistical environment (R Foundation for Statistical Computing, Vienna, Austria). The MHC diversity and microsatellite heterozygosity were entered as response variables in separate GLMMs, while population was entered as a four-level fixed factor. In the analysis of the MHC we also added microsatellite heterozygosity as a covariate to control for neutral genetic variation. The effect of year was included in both models as a random factor to control for inter-annual variation.

In order to assess genetic differentiation between populations, we used two major approaches. First, we calculated pairwise Jost’s D measures of differentiation for both MHC and microsatellite data (Jost, 2008). Calculations of Jost’s D are based on the effective number of alleles (Jost, 2008), instead of expected heterozygosity, as in the case of F_ST_ and similar statistics (Meirmans & Hedrick, 2011), which was unknown for multilocus MHC data. Jost’s D estimates for MHC data were calculated in the *SpadeR* R package (Chao et al., 2016) and for the purpose of analyses each allele was coded as present or absent (as in a dominant marker). For microsatellite data, Jost’s D values were estimated using bootstrapping (1,000 permutation) in the *diveRsity* R package (Keenan et al., 2013). Additionally, we calculated standardized G’_ST_ values (*i.e*., pairwise G_ST_ values divided by the maximum G_ST_ value; Hedrick, 2005), but this was done exclusively for microsatellite loci. A correlation between pairwise Jost’s D values for MHC and microsatellites was tested using Pearson product-moment correlation coefficient.

Second, we used Bayesian clustering algorithm implemented in the program STRUCTURE (Pritchard, Stephens & Donnelly, 2000) to infer the number of genetic clusters (*K*) in the data and to assign individual genotypes to these clusters (separately for MHC and microsatellite data). Since it was not possible to assign MHC alleles to loci, we encoded each allele as a dominant biallelic locus, following Herdegen, Babik & Radwan (2014) and ran the analysis of MHC data as for dominant markers (Falush, Stephens & Pritchard, 2007). We set the number of tested *K* values between one and five. We used the admixture model of ancestry, correlated model of allele frequencies, and sampling location as prior information. The number of Markov chain Monte Carlo iterations was set to 5,00,000 and the length of burn-in period was set to 2,00,000. Each analysis was replicated ten times. The number of genetic clusters for each type of marker was inferred by a comparison of mean posterior probabilities for different *K* values L(*K*) (also referred to as likelihood scores), as implemented in the Structure Harvester 0.6.94 (Earl & von Holdt, 2012). We preferred L(*K*) against the Δ*K* algorithm (Evanno, Regnaut & Goudet, 2005), since the latter does not test for *K* = 1 and, thus, it may overestimate the number of genetic clusters under the homogeneous genetic structure (Janes et al., 2017). Multiple runs of clustering analysis were averaged with Clumpp 1.1.2 (Jakobsson & Rosenberg, 2007) and the final output was visualized with Distruct 1.1 (Rosenberg, 2004). All values are reported as means ± SE.

## Results

### MHC and microsatellite polymorphism

After clustering and filtering of Illumina sequences, we found that 115 MHC class II alleles were retained across all populations. These corresponded to 113 unique amino acid sequences. Reproducibility of alleles after processing was 100% (see File S2 for allele calling across 25 technical replicates). Between one and six alleles were recorded per individual, being consistent with the presence of three MHC class II loci in the Eurasian coot. Most individuals (53.4%, *n* = 103) had three MHC alleles and the mean number of MHC alleles was 2.89 ± 0.08 per individual.

Most microsatellite loci showed moderate polymorphism (3–17 alleles per locus), except for Kira-16, where 48 alleles were recorded (Table S1). The observed heterozygosity (Ho) of microsatellites was 0.59–0.66 per population (Table S3) and 0.09–0.88 per locus (Table S1).

### Reduced MHC diversity in the old urban population

**Figure 2:**
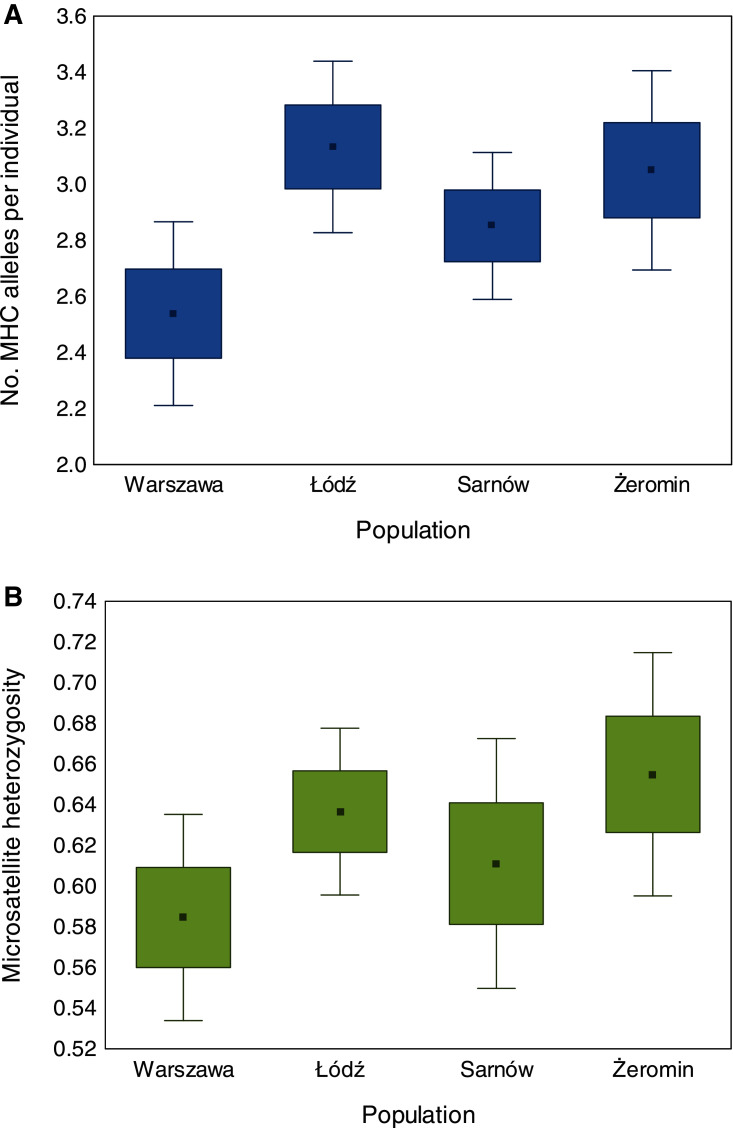
Differences in the number of MHC class II alleles per individual (A) and individual heterozygosity at microsatellite loci (B) between old urban (Warszawa), new urban (Łódź) and two rural (Sarnów and Żeromin) populations of the Eurasian coot. Statistical significance for pairwise comparisons is reported in Table 2.

**Figure 3:**
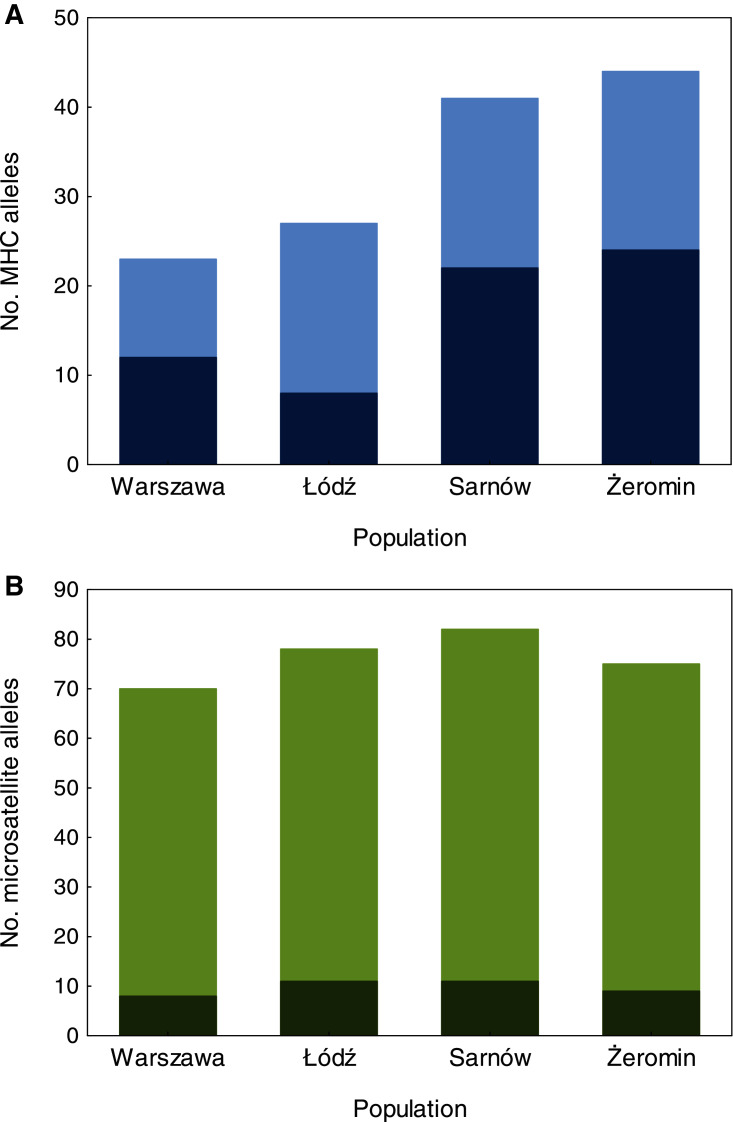
The number of MHC class II (A) and microsatellite (B) alleles recorded in old urban (Warszawa), new urban (Łódź) and two rural (Sarnów and Żeromin) populations of the Eurasian coot. All estimates are shown for a standardized population size of 20 individuals. Dark colours represent private (population-specific) alleles, while light colours represent alleles shared between populations.

We found significant differences in individual MHC diversity (*i.e*., the number of MHC alleles per individual) between the populations (Fig. 2), as MHC diversity was significantly lower in the old urban population (Warszawa), when compared with the new urban population (Łódź; β = −0.60 ± 0.21, *P* = 0.005, Table 1) and one of the rural populations (Żeromin; β = −0.52 ± 0.23, *P* = 0.029, Table 1). There were no significant differences between the new urban population and both rural populations (Table 1). Rural populations were characterized by the highest total number of MHC alleles, as well as the highest number of private MHC alleles (Table S2). A similar pattern was revealed after the standardization of the sample sizes between populations, showing that the total number of MHC alleles was 34–48% lower in urban than rural populations (Table S2, Fig. 3A). Despite higher allelic diversity in rural populations, all measures of MHC sequence polymorphism (*i.e*., number of segregating sites, number of nucleotide differences, and nucleotide diversity) were similar across urban and rural populations (Table S2). In contrast to the MHC, we found no differences in the individual heterozygosity at the microsatellite loci between the populations (Table 1; Fig. 2), although the difference between the old urban population (Warszawa) and one of the rural populations (Żeromin) was only marginally non-significant (lower heterozygosity in Warszawa; β = −0.070 ± 0.039, *P* = 0.072, Table 1). The total number of alleles and the number of private alleles at microsatellite loci showed little variation across all four populations (Table S3, Fig. 3B).

**Table 1:** Differences in the individual MHC diversity (the number of MHC class II alleles per individual) and individual microsatellite heterozygosity between populations.

Marker	Predictor	Estimate ± SE	t	*P*
MHC class II	**Intercept**	**2.58 ± 0.38**	**6.82**	**<0.001**
	**Population (Warszawa *vs*. Łódź)**	**−0.60 ± 0.21**	**−2.87**	**0.005**
	Population (Warszawa *vs*. Sarnów)	−0.32 ± 0.21	−1.49	0.14
	**Population (Warszawa *vs*. Żeromin)**	**−0.52 ± 0.23**	**−2.22**	**0.029**
	Population (Łódź *vs*. Sarnów)	0.28 ± 0.21	1.38	0.17
	Population (Łódź *vs*. Żeromin)	0.08 ± 0.22	0.37	0.71
	Population (Sarnów *vs*. Żeromin)	−0.20 ± 0.22	−0.88	0.38
	Microsatellite heterozygosity	−0.08 ± 0.59	−0.14	0.89
Microsatellites	**Intercept**	**0.584 ± 0.026**	**22.89**	**<0.001**
	Population (Warszawa *vs*. Łódź)	−0.052 ± 0.035	−1.49	0.14
	Population (Warszawa *vs*. Sarnów)	−0.026 ± 0.036	−0.74	0.46
	Population (Warszawa *vs*. Żeromin)	−0.070 ± 0.039	−1.82	0.072
	Population (Łódź *vs*. Sarnów)	0.026 ± 0.034	0.74	0.46
	Population (Łódź *vs*. Żeromin)	−0.018 ± 0.038	−0.49	0.63
	Population (Sarnów *vs*. Żeromin)	−0.044 ± 0.038	−1.14	0.26

**Notes:**

(Warszawa–old urban population, Łódź–new urban population, Sarnów and Żeromin–rural populations). Year was included as a random factor.

Significant predictors are marked in bold.

**Table 2:** Genetic differentiation between urban and rural Eurasian coot populations at the MHC and microsatellite loci, as measured with pairwise Jost’s D (below diagonal) and G’_ST_ (above diagonal) values.

Marker	Population	Warszawa (old urban)	Łódź (new urban)	Sarnów (rural)	Żeromin (rural)
MHC class II	Warszawa	–	NA	NA	NA
	Łódź	**0.602**	–	NA	NA
	Sarnów	**0.588**	**0.277**	–	NA
	Żeromin	**0.623**	**0.413**	0.142	–
Microsatellites	Warszawa	–	0.019	0.009	0.025
	Łódź	0.012	–	0.020	0.020
	Sarnów	0.005	0.006	–	0.019
	Żeromin	0.017	0.018	0.005	–

**Note:**

Significant comparisons (*P* < 0.05) are indicated in bold.

### Significant MHC differentiation between urban and rural populations

We found strong population differentiation at the MHC and the average pairwise Jost’s D was 0.441. The strongest differentiation at MHC genes was recorded between the old urban population (Warszawa) and all other populations, although the new urban population (Łódź) also showed significant differentiation from both rural populations (Table 2). The two rural populations showed no significant differentiation at the MHC (Jost’s D = 0.142 ± 0.152; Table 2). No significant population differentiation was found at the microsatellite loci, as measured with Jost’s D and G’_ST_ (Table 2). Pairwise Jost’s D values for MHC and microsatellites did not show significant correlation (r = 0.47, *n* = 6, *p* = 0.35).

### MHC data suggests two genetic clusters

**Figure 4:**
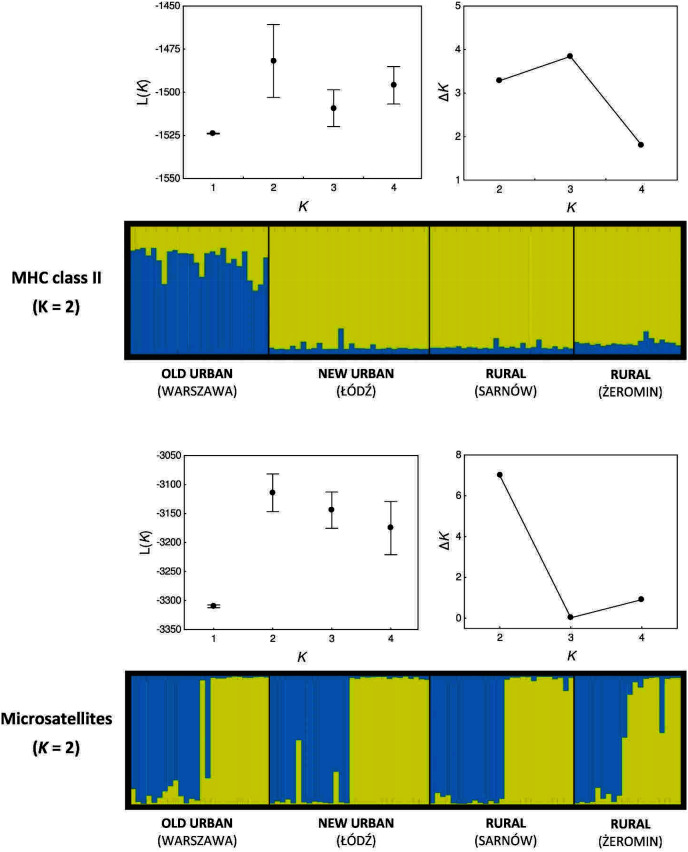
Bayesian assignment of Eurasian coots from two urban and two rural populations to genetic clusters. Each bar represents the estimated proportional posterior probability of each individual belonging to each cluster, as marked with different colours. Genetic clusters were inferred separately for MHC class II genes and ten microsatellite markers. Mean (±SD) posterior probability L(K) and Δ*K* values for different number of clusters (*K*) are shown above each assignment plot.

Our Bayesian assignment of individuals to genetic clusters also provided support for greater MHC differentiation between populations (when compared with microsatellites). Two genetic clusters were inferred for the MHC genes, as based on the mean posterior probabilities L(*K*), although Δ*K* were similar for *K* = 2 and *K* = 3 (Fig. 4). All individuals from the new urban and both rural populations were assigned to a single genetic cluster with very high assignment probabilities (0.933 ± 0.002; yellow cluster, Fig. 4). The old urban population clustered exclusively to itself, indicating differentiation from the remaining populations (blue cluster, Fig. 4), although average assignment probabilities to this cluster were only moderately high (0.738 ± 0.020). Two genetic clusters were also inferred for microsatellite loci, as indicated by both L(*K*) and Δ*K* values (Fig. 4). The pattern was highly contrasting with the MHC, as a similar proportion of individuals from each population was assigned to each cluster, providing no support for neutral genetic differentiation between populations (Fig. 4).

## Discussion

Our study investigated MHC class II gene divergence between Eurasian coots sampled in urban and non-urban environments, where different pathogen pressure may occur. We found significant differentiation of MHC genes between both urban populations and the populations inhabiting non-urban areas. We also demonstrated that the strongest differentiation of MHC genes occurred between the old urban population (Warszawa) and the three others-the new urban (Łódź) and two non-urban populations (Sarnów and Żeromin). Finally, there was an apparent loss in MHC allele diversity in both urban populations when compared to rural ones. These findings were not consistent with the patterns of neutral genetic diversity and differentiation between the populations (across a panel of microsatellite loci), suggesting that processes shaping MHC variation were not primarily driven by demographic processes.

MHC genes are an important model for studying the adaptive genetic differentiation in wildlife populations, as co-evolution with pathogens is commonly referred to as the main mechanism responsible for the occurrence of MHC polymorphism (Spurgin & Richardson, 2010). Our results are suggestive of adaptive differentiation at the MHC in urban populations, supported by the lack of genetic differentiation at neutral loci. These results were inconsistent with our previous research, showing significant microsatellite differentiation of urban and non-urban coots in Poland (Minias et al., 2017). Yet, it is important to note that our earlier analyses were conducted on a larger number of populations using a directly paired (urban *vs*. adjacent non-urban) framework. This was possible due to the other type of material collected (*i.e*., moulted feathers), which did not allow efficient MHC genotyping because of low quality DNA extracts. Here, we used blood sampled from captured individuals, which on one hand allowed us to obtain reliable MHC genotypes, but on the other hand it limited our sampling and reduced statistical power to detect inter-population differentiation. It must be acknowledged, however, that the results on microsatellite and MHC variation within this study were obtained under identical sampling scheme and, thus, can be reliably compared. Taking this into account, contrasting patterns for neutral genetic variation (a lack of differentiation) and adaptive immunogenetic variation (a significant differentiation at the MHC) suggest that the latter cannot be solely explained by stochastic mechanisms associated with genetic drift or demographic processes, which may take place during urban colonization.

The urbanization process may be associated with a random reduction in genetic diversity as a result of the bottleneck effect (*i.e*., when a group of individuals colonizing novel areas is genetically not representative for the source population; Evans et al., 2010). Traces of such processes should be, however, detected both at the level of neutral and adaptive genetic markers, which was not confirmed in our study. Not only was there no significant differentiation in microsatellite loci found between our coot populations, but we also observed little differences in the diversity of these loci between the populations. The only pairwise difference in microsatellite heterozygosity that was close to the significance threshold indicated a tendency for a lower heterozygosity in the old urban populations when compared to a non-urban one. This may suggest that demographic processes, such as recent genetic bottleneck or founder effect, could have contributed to the patterns of genetic variation that we observed between our study coot population, but this contribution was rather low and unlikely to explain strong differentiation at the MHC genes. Alternatively, MHC alleles may show lower average frequencies within populations when compared to microsatellites and, for this reason, they may be more prone to loss during urban colonization processes, which can promote stronger differentiation at the MHC between urban and non-urban populations. However, it was unfeasible to calculate true frequencies of MHC alleles without locus-specific data and, thus, we were unable to directly test this hypothesis.

Our results suggest that MHC differentiation between populations could stem from the processes acting specifically on these genes. The processes of urban colonization by wildlife may require adaptations of key immune receptors to the composition of local pathogen and parasite faunas. Congruent with this hypothesis, our analyses showed differences in the MHC allele composition between populations that differed in the level of urbanization. There are several non-exclusive mechanisms which may explain this pattern, although we could not effectively distinguish between these alternative scenarios with our data. First, differences in the allelic composition of MHC between the new urban population (Łódź) and the non-urban populations (putative source populations for the colonization of Łódź; Minias et al., 2017) may suggest that the influx on non-urban individuals into cities could have proceeded through the sorting of MHC genotypes. This mechanism assumes that only individuals with specific MHC genotypes, pre-adapted to the urban pathogen fauna, can effectively settle, survive and raise offspring in the urban environment. Similar mechanisms are observed at the phenotype level, where birds with particular behavioural features (*e.g*., more aggressive, bolder, more explorative or more resistant to stress) are more likely to colonize novel habitats, including urban areas (Luniak, 2004; Evans et al., 2010; Minias, Jedlikowski & Włodarczyk, 2018). However, it must be stressed that our study did not directly test this hypothesis and further research into the potential for genotype sorting is required.

Alternatively, differentiation at the MHC between the old and new urban populations may suggest the occurrence of local adaptations, where MHC repertoire becomes gradually adapted to the local pathogen pressure (Lan et al., 2019). In accordance with our results, the strength of the local MHC adaptations in urban populations should be correlated with the time of the exposure of a given population to the urban pathogen fauna (under similar strength of pathogen-driven selection in both populations), and, therefore, indirectly with the time at which a given urban population was established. At the same time, the old urban population showed the lowest individual MHC variability (*i.e*., the average number of alleles per individual). This may suggest an adaptation to a specific (perhaps relatively homogenous) assemblage of urban pathogens and it is consistent with empirical studies showing altered composition of parasite faunas in an urbanized landscape and a negative association of parasite richness with urbanization level (Calegaro-Marques & Amato, 2014; Łoś et al., 2020). We, however, explicitly acknowledge that no pathogen data were collected in our study, and we cannot draw any firm conclusions with this respect. Also, we cannot rule out that this effect can simply have arisen due to different strength of pathogen-driven selection in each environment and may not be directly associated with the timing of population establishment.

Our capability to infer the exact mechanisms responsible for the observed MHC differentiation was further impaired by some methodological limitations. First, we had no replicates for old and new urban populations and, thus, our analyses yielded no statistical power to directly test for the effect of population history (*i.e*., timing of urban colonization) on MHC differentiation. Also, our populations were not evenly distributed in space, as the old urban population was located relatively far from the remaining three (new urban and two rural) populations. This may have suggested that any genetic differentiation of the old urban population could have arisen due to isolation by distance, although this scenario has not been confirmed by the analysis of microsatellite data (*i.e*., the lack of significant differentiation between populations).

In general, maintenance of local adaptations may be facilitated by limited gene flow between populations. For example, research on burrowing owls (*Athene cunicularia*) has shown that there is limited gene flow between adjacent urban and rural populations (*i.e*., enhancing local adaptation), but not between different urban populations (Mueller et al., 2018). Basic factors that can reduce gene flow between urban and non-urban populations include marked differences in habitat structure and intensity of anthropogenic pressure, but also differences in local climate conditions or predatory pressure (*e.g*., in the composition of predator faunas; Edelaar, Siepielski & Clobert, 2008). For instance, the shift from high genotypic variation in rural landscapes to strongly favoured genotypes in urban settings was observed in wood frogs (*Lithobates sylvaticus*), as a results of strong habitat differences which diminished gene flow and amplified genetic drift (Homola et al., 2019). This habitat variation is often reflected by high behavioural divergence between urban and non-urban individuals (Shochat, 2004), which may further reduce migration rate of individuals (genes) across landscapes. Indeed, behavioural studies conducted in our study coot populations revealed different spectra of behaviours between the urban and non-urban birds. It was demonstrated that coots from the old urban population (Warszawa) were more aggressive while defending the nest, less sensitive to stress and the presence of humans, and used food of anthropogenic origin better than individuals from the new urban population (Łódź) and the non-urban populations (Minias, Jedlikowski & Włodarczyk, 2018). It seems that this kind of behavioural differentiation may constitute one of the mechanisms promoting the maintenance of local genetic adaptations (also at the level of MHC) in the urban areas. On the other hand, an increasing number of studies show that local adaptations can be maintained despite high gene flow (reviewed in Tigano & Friesen, 2016). Preservation of local adaptations under gene flow is facilitated by several genomic processes (*e.g*., rearrangements of genomic architecture, linkage with already diverged loci, or mechanisms suppressing recombination) that maintain clusters of adaptive loci (Tigano & Friesen, 2016). Since our analyses of neutral (microsatellite) genetic variation indicated no apparent barriers for gene flow between our coot populations, it seems possible that some of these mechanisms could contribute to the maintenance of local adaptations at the MHC genes. So far, evidence of local adaptation to urbanization in the face of gene flow was found in a highly mobile insect pollinator, the red-tailed bumblebee (*Bombus lapidarius*; Theodorou et al., 2018).

In conclusion, our study showed significant between-population differentiation of MHC repertoire depending on the level of urbanization, likely as a result of non-neutral processes (genotype sorting or local adaptation). Currently, there is a scarcity of studies testing for immunogenetic adaptations to urban habitats in birds and our results provide an important contribution to this topic, as well as advancing our more general understanding of genetic mechanisms associated with urbanization processes in wildlife. Although our results revealed associations of MHC allele composition with habitat urbanization and the time which passed since urban colonization, we are aware that our conclusions based on a limited number of populations should be confirmed using longitudinal data collected at broader geographical scale and across a larger number of urban and non-urban population replicates. We also acknowledge that future research should complement our findings with pathogen data from different habitats and, ideally, should examine fitness consequences associated with different MHC composition in urban and non-urban environments. Nevertheless, we believe that our study provides important foundational information that future work can build upon, not only in coots, but also pertaining to other urban colonizing wildlife. As our knowledge of how species are evolutionarily responding to increasing rates of urbanization grows, knowing the role immunogenetic adaptations play will provide key insights into urbanized taxa and what it takes to survive in a human world.

## Supplemental Information

10.7717/peerj.12264/supp-1 Supplemental Information 1 Microsatellite loci used to assess neutral variation and diversity measures in the Eurasian coot.

10.7717/peerj.12264/supp-2 Supplemental Information 2 AmpliSAS results for techincal replicates, showing the number of Illumina reads per allele (after processing).

10.7717/peerj.12264/supp-3 Supplemental Information 3 Microsatellite and MHC genotypes (raw data).

## Additional Information and Declarations

### Field Study Permissions

The following information was supplied relating to field study approvals (*i.e*., approving body and any reference numbers): Field experiments were approved by the Local Bioethical Commission for Experiments on Animals in Łodź (nos 40/ŁB 620/2012 and 15/ŁB/2016). Access to non-urban sites was granted by private land owners (Agricultural and Fishing Farms at Sarnów and Żeromin).

### DNA Deposition

The following information was supplied regarding the deposition of DNA sequences: All novel MHC sequences are available in GenBank: MT74863–MT748786. All other alleles were previously described by Alcaide et al. (2014).

### Data Availability

The following information was supplied regarding data availability: The raw genetic data (MHC and microsatellites) are available in the Supplementary File.
