# Supplementary material for: Urbanization processes drive divergence at the major histocompatibility complex in a common waterbird
Source: PeerJ. 2021 Oct 5;9:e12264. doi: 10.7717/peerj.12264 (PMC8500109; doi:10.7717/peerj.12264)
Supplement: Supplemental Information 1 [file peerj-09-12264-s001.docx]

**Supplementary File 1**

**Urbanization processes drive divergence at the Major Histocompatibility Complex in a common waterbird**

**Ewa Pikus^1^, Radosław Włodarczyk^1^, Jan Jedlikowski^2^, Piotr Minias^1^**

^1^ Department of Biodiversity Studies and Bioeducation, Faculty of Biology and Environmental Protection, University of Łódź, Banacha 1/3, 90-237 Łódź, Poland

^2^ Faculty of Biology, Biological and Chemical Research Centre, University of Warsaw, Żwirki i Wigury 101, 02-089 Warsaw, Poland

Corresponding author:

Piotr Minias, pminias@op.pl

**Table S1** Microsatellite loci used to assess neutral variation in the Eurasian coot. Repeat unit size, size range, number of alleles, observed heterozygosity (H_o_) and expected heterozygosity (H_e_) are reported for each marker.

| Locus | Forward primer  sequences (5’–3’) | Reverse primer  sequences (5’–3’) | Repeat unit size (bp) | Size range (bp) | N alleles | H_o_ | H_e_ |
| --- | --- | --- | --- | --- | --- | --- | --- |
| Tm18 | CTGAACATTTCAAGAGTCCTCA | GCAGAGCTGACCACTCAGAAC | 2 | 137-149 | 3 | 0.703 | 0.539 |
| Tm20 | TGCACAGATGAGAAGGAGGGC | GCCTGCTCACACTAGCAGAAC | 2 | 127-133 | 3 | 0.090 | 0.085 |
| Tm27 | CTAGGTCACCTTTACAGTAGTC | CAATACCATGTATAGAAATGTTGG | 2 | 131-173 | 17 | 0.836 | 0.846 |
| Tm38 | GATAGATGGAGCGTAAAATGCA | AAGAAAGCAAAATGTCTGTATTGG | 2 | 103-113 | 6 | 0.504 | 0.511 |
| Kira8 | GCTTTGCATCTTGCCTTAAA | GTGACACTGATACAGTGTGCCT | 2 | 307-331 | 16 | 0.879 | 0.901 |
| Kira9 | TGATCTGGGCAGGCTTCTAC | GTCGAATAATGGCAGCAATG | 2 | 163-173 | 5 | 0.520 | 0.499 |
| Kira10 | CCAAGTACCATCTGCGAAGC | AACCCGAACGAGAGATGTGA | 2 | 121-145 | 12 | 0.785 | 0.774 |
| Kira16 | CCAGGTGAAACTCTGCATT | ACAGTTGTGATGTGGCTGGA | 2 | 299-419 | 48 | 0.858 | 0.930 |
| B106 | TAGTGCTCTCAGGAAAGACTTG | CTCTTCCAGAAGCTGTAGTTG | 2 | 168-204 | 15 | 0.777 | 0.788 |
| TG03002 | TCTTGCCTTTTTGGTATGAGTAT | TACAAAGCACTGTGGAGCAG | 2 | 118-126 | 5 | 0.265 | 0.257 |

**Table S2** Measures of MHC class II diversity in old urban (Warszawa), new urban (Łódź) and two rural (Sarnów and Żeromin) populations of the Eurasian coot. Estimates given in parentheses were standardized for a population size of 20 individuals using rarefaction.

| Population | No. individuals | No. alleles | No. private alleles | No. segregating sites | No. nucleotide differences | Nucleotide diversity |
| --- | --- | --- | --- | --- | --- | --- |
| Warszawa | 26 (20) | 27 (23) | 14 (12) | 93 (90) | 32.4 (32.5) | 0.121 (0.122) |
| Łódź | 30 (20) | 37 (27) | 14 (8) | 93 (90) | 31.2 (32.0) | 0.117 (0.119) |
| Sarnów | 27 (20) | 52 (41) | 30 (22) | 96 (93) | 31.3 (32.5) | 0.117 (0.122) |
| Żeromin | 20 (20) | 44 (44) | 24 (24) | 92 (92) | 31.4 (31.4) | 0.118 (0.118) |

**Table S3** Measures of microsatellite diversity in old urban (Warszawa), new urban (Łódź) and two rural (Sarnów and Żeromin) populations of the Eurasian coot. Estimates given in parentheses were standardized for a population size of 20 individuals using rarefaction.

| Population | No. individuals | No. alleles | No. private alleles | Observed heterozygosity (H_o_) |
| --- | --- | --- | --- | --- |
| Warszawa | 26 (20) | 76 (70) | 8 (8) | 0.585 (0.580) |
| Łódź | 30 (20) | 88 (78) | 13 (11) | 0.637 (0.655) |
| Sarnów | 27 (20) | 88 (82) | 13 (11) | 0.611 (0.635) |
| Żeromin | 20 (20) | 75 (75) | 7 (9) | 0.655 (0.655) |
